# Long-term Outcome of Recoverable stents for Budd-Chiari syndrome Complicated with Inferior Vena Cava Thrombosis

**DOI:** 10.1038/s41598-018-25876-w

**Published:** 2018-05-09

**Authors:** Yonghua Bi, Hongmei Chen, Penxu Ding, Jianzhuang Ren, Xinwei Han

**Affiliations:** 1 Department of Interventional Radiology, The First Affiliated Hospital of Zhengzhou University, Zhengzhou, China; 2 Department of Ultrasound, Zhengzhou Central Hospital Affiliated to Zhengzhou University, Zhengzhou, China

## Abstract

This study aimed to present long-term results of a 12-year patient follow-up of recoverable stents for BCS complicated by inferior vena cava (IVC) thrombosis. Forty consecutive patients with BCS complicated by IVC thrombosis were treated with recoverable stents. The median duration of symptoms was 24 months. Recoverable stents was placed after predilation of the obstructed IVC, and then agitation thrombolysis or catheter-directed thrombolysis of IVC was performed. The recoverable stents was removed eventually after thrombus disappeared. Clinical patency was defined as absence or improvement of symptoms. Patients were subsequently followed-up by color Doppler ultrasound. Recoverable stents placement, balloon angioplasty and thrombolysis were technically successful in all patients. Stents were successfully removed in 92.1% of patients. A few serious related complications including one acute pulmonary thromboembolism, one stent migration, and one failure retrieval stents occurred. The median follow-up was 43.7 months. The long-term results were satisfactory except 2 patients who presented with a restenosis or re-obstruction and underwent additional therapy. There were 5 deaths owing to pulmonary embolism or underlying malignant disease 0.4–101.8 months after the procedures, including one procedure-related death. In conclusion, Recoverable stents treatment is safe and effective for BCS complicated by IVC thrombosis, with a good long-term outcome.

## Introduction

Budd-Chiari syndrome (BCS) is a rare disease in Western countries and most patients commonly present with pure hepatic veins involvement^1,2^. However, the prevalence of BCS is higher in Asia, and most BCS patients have complex lesions combining inferior vena cava (IVC) and hepatic vein involvement^3^, which is considered the most common etiological factor. Treatment is also different, transjugular intrahepatic portosystemic stent shunt is the preferred treatment and liver transplantation is often needed in Western countries. In Asia, balloon angioplasty and stenting is the first choice rather than liver transplantation^4–7^, with a better long-term prognosis^8,9^. Balloon angioplasty or stent placement has been considered successfully in treating IVC obstruction^4,7^. However, treatment of IVC thrombosis remains challenging in BCS patients, which precludes balloon angioplasty or stents due to the risk of acute pulmonary thromboembolism. Although most patients can been successfully managed with a variety of treatments, including local thrombolysis, angioplasty, and stents^10^, restenosis still occurs in approximately 13% of BCS patients after stent placement secondary to thrombus^7^. Follow-up by Doppler ultrasonography is needed to determine the patency of IVC^7,11,12^. The recoverable stents may beneficial to avoid long-term complications of permanent stents, including stent migration, restenosis or occlusion of IVC, and so forth. This study aims to present long-term results of a 12-year patient follow-up of recoverable stents for BCS complicated by IVC thrombosis.

## Materials and Methods

### Study design

**Figure 1:**
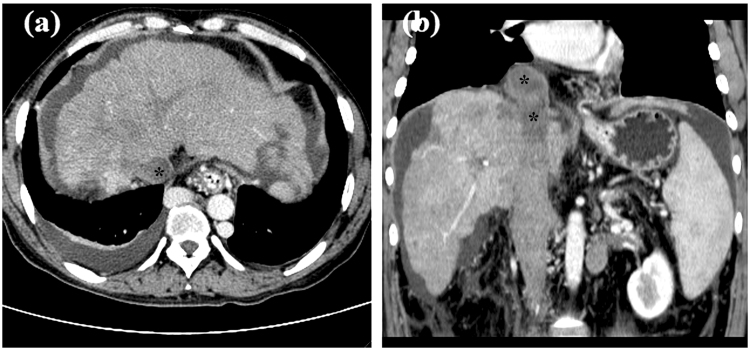
Preoperative CT scanning. (**a**) Liver cirrhosis caused by BCS, hydrothorax, and can be seen in the cross section of CT. (**b**) Segmental occlusion of IVC with thrombus was shown in coronal section view. * indicated thrombus.

Between December 2003 and August 2016, 40 consecutive patients with BCS complicated by IVC thrombosis were treated with recoverable stents in our department. Patients were chosen for the study based on a review of the patient’s history and findings of color Doppler sonography, CT scanning, and IVC angiography (Fig. 1). Patients were followed-up by color Doppler ultrasound in the outpatient department. This retrospective study was approved by Zhengzhou university committee on human investigation and written informed consent was obtained from all patients. All methods were performed in accordance with the relevant guidelines and regulations.

### Data availability

No datasets were generated or analysed during the current study.

### Thrombolysis

**Figure 2:**
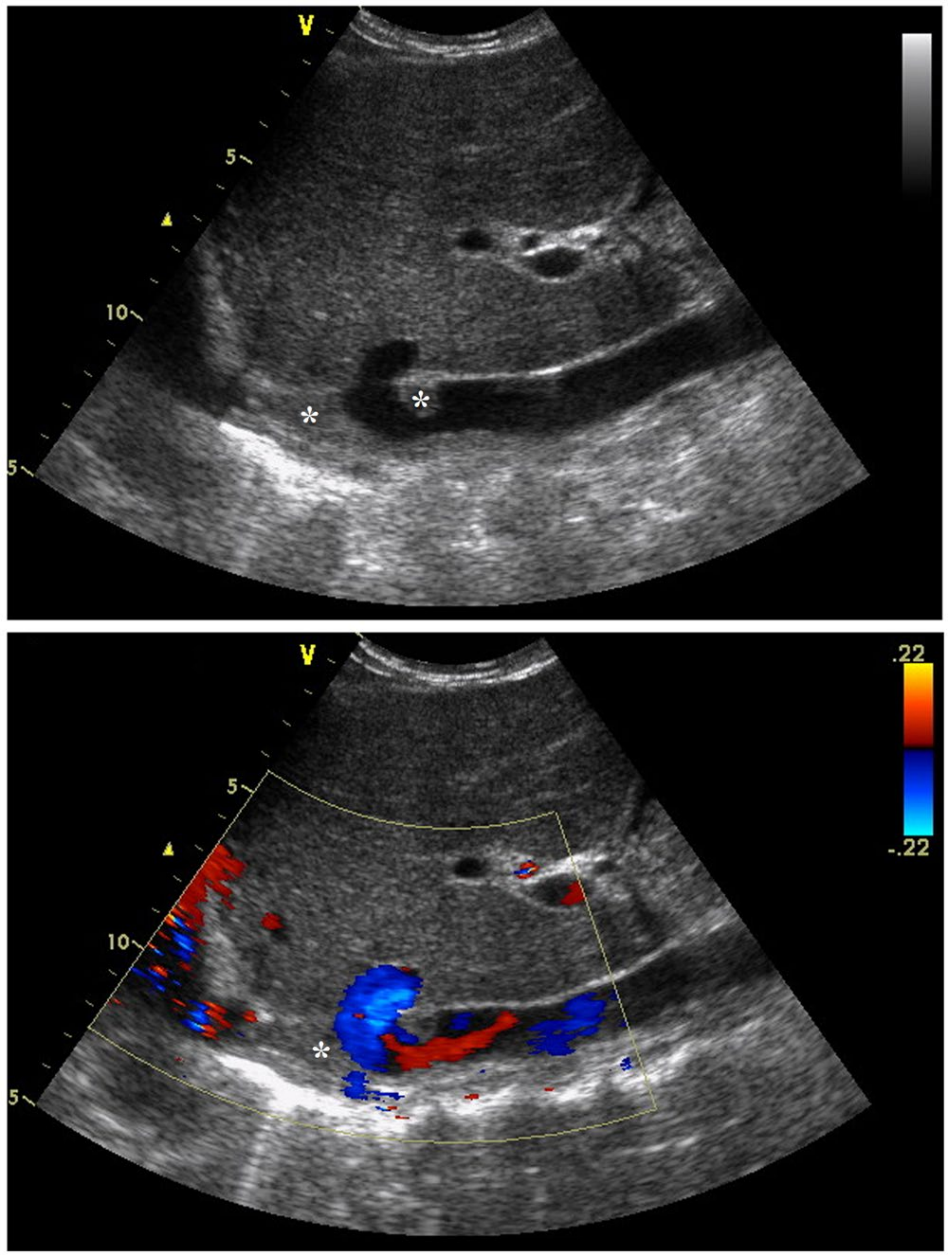
Preoperative color Doppler sonography. Segmental occlusion, thrombus and a small amount of blood flow echoes were shown in IVC. * indicated thrombus.

Thrombolysis was performed in patients with acute thrombosis, whose lesion showed low echo in Doppler sonography (Fig. 2). All procedures were performed under local anesthesia. A 5-Fr catheter with multiple side holes (Cook, Bloomington, IN, USA) was introduced along a guide wire and positioned below the occluded segment of IVC. At the beginning of the procedure, IVC angiography was performed to show the occluded segment of IVC, and the location and size of thrombus. Agitation thrombolysis of IVC, a catheter and guide wire with a helical tip configuration alternatively rotated or pushed and drawn, was used to agitate the thrombosis, and/or catheter-directed thrombolysis by continuous infusion of urokinase at a rate of 10–20 × 10^5^ U/h for 2 hours was then performed to dissolve the fresh thrombus. Patients took warfarin orally and received subcutaneous injections of low-molecular-weight heparin (5100 IU every 12 h) for several days, until the International Normalized Rate (INR) reaches 2–3. Then only warfarin was taken for anticoagulation, and a longer (>12 months) period was necessary for patients with comorbidities of renal vein thrombosis or lower limb venous thrombus.

### Predilation and postdilation

A balloon catheter with a diameter of 12–16 mm was introduced along a stiff guide wire and predilated at the site of the obstructed segment to restore luminal patency of IVC. Postdilation was performed after removal of the recoverable stents if IVC stenosis persisted. The stenosed segment was dilated 1 to 3 times with a 25, 26 or 30-mm balloon catheter during interventional procedure. IVC angiography was performed immediately after recoverable stents removal and postdilation to confirm patency of IVC.

### Recoverable stents placement and retrieval

**Figure 3:**
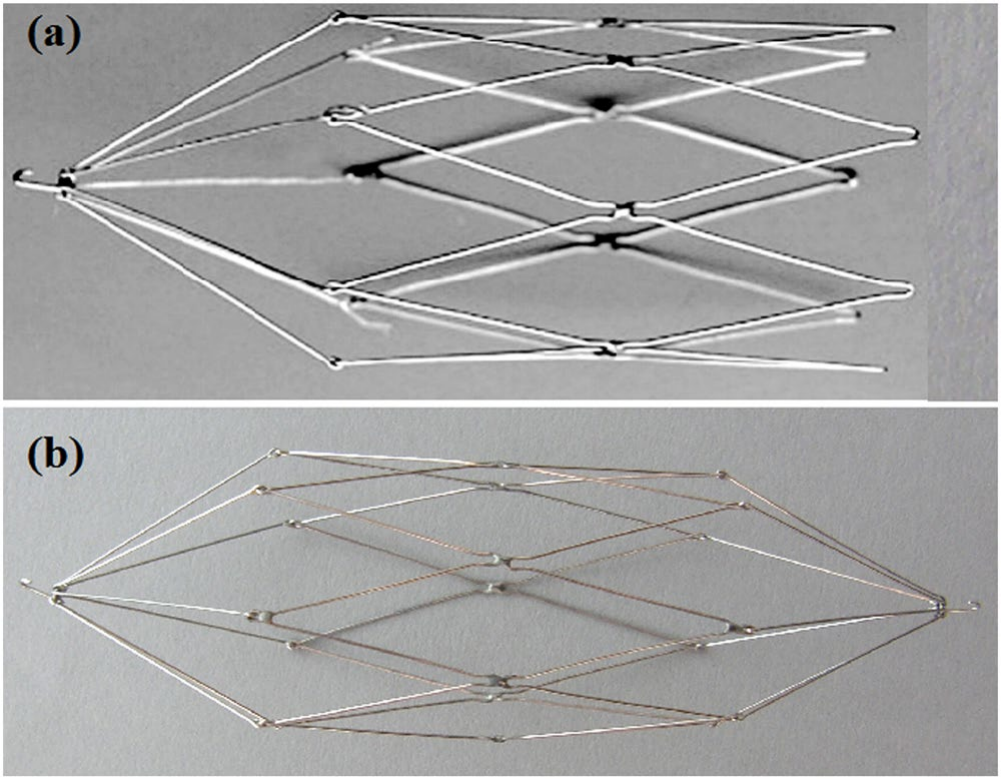
Photograph of the recoverable stents. The recoverable stents consists of seven legs with one (**a**) or two (**b**) retrieval hooks at the apex of the cone lay.

**Figure 4:**
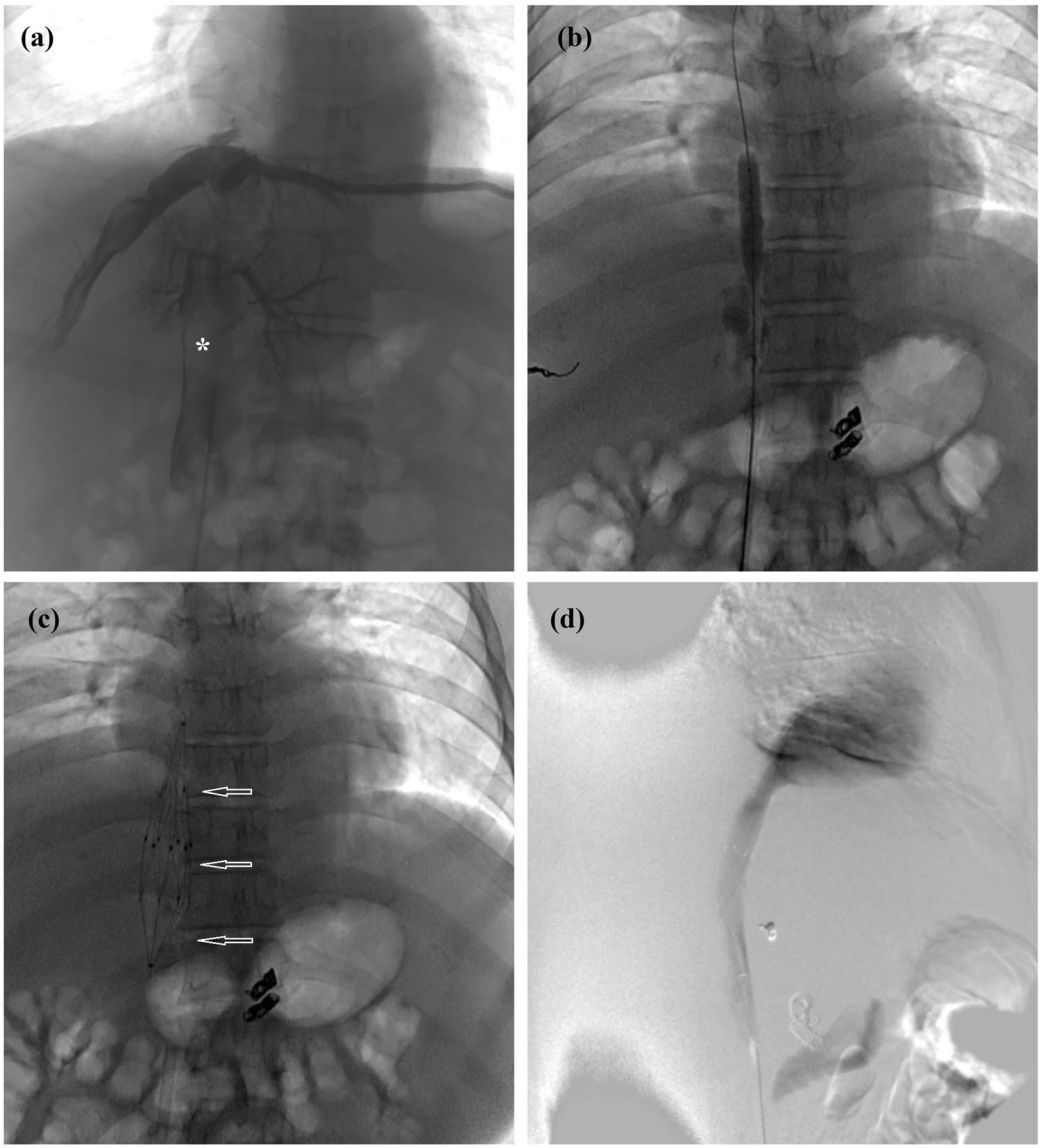
Predilation with small balloon and stent implantation. (**a**) Segmental occlusion, thrombus (*) was confirmed by angiography. (**b**) Predilation performed with a 10-mm balloon. (**c**) A stent of 30 mm in diameter, and 108 mm in length (arrows) was implanted. (**d**) Second angiography showed a patency of IVC after stenting.

A same type of stent was used through out the study period. Recoverable stents (Yong-Tong Co, Shenyang, China) consists of seven legs, with a maximum diameter of 30 mm and length of 54 mm or 108 mm. A retrieval hook lies at the apex of the cone, which was attached to the seven legs by a 2.5-mm loop (Fig. 3). Recoverable stents were introduced with the Z-stent positioned across the occluded segment of IVC and deployed by withdrawing the sheath. IVC angiography was performed immediately to verify whether the location of the stents and blood flow was satisfactory (Fig. 4). Recoverable stents were retrieved, similar to an IVC filter, via a transjugular or transfemoral approach if complete disappearance of the thrombus was confirmed by color Doppler ultrasonography. An Amplatz gooseneck snare (MicroVena, White Bear Lake, MN, USA) was introduced through a 14-Fr introducer sheath to capture and then withdraw the recoverable stents. IVC angiography was performed again to show the final status of IVC and possible complications.

### Perioperative observation and postoperative follow-up

Vital signs and perioperative complications were monitored during thrombolysis. Color Doppler ultrasound was performed to monitor thrombosis disappearance, such as length and thickness of thrombus. The diameter of IVC, speed of blood flow and patency of IVC were studied during perioperative observation and postoperative follow-up. Complications related to the procedures, mortality, morbidity, and final clinical outcome were collected prospectively. Major complications were defined as death, massive bleeding from IVC, and fracture, migration or insufficient expansion of recoverable stents. Technical success was defined as successful thrombolysis, balloon dilation, recoverable stents placement and removal without any complications. Primary patency was defined as the interval between initial treatment and the first appearance of re-obstruction or restenosis of IVC. Secondary patency was defined as patency after the initial treatment until all attempts at maintaining patency were abandoned.

### Statistical analysis

Data are presented as the mean ± SD or median with range. One way ANOVA was used. The *p*-value was considered statistically significant if *p* < 0.05.

## Results

### Patient population

The 40 consecutive patients (32 men and 8 women) had a median age of 49.5 years (range: 28–76 years). The duration of symptoms ranged from 0.1 to 480 months (median 24, IQR 1.2, 123 months). For lesion types of BCS, 37 (92.5%) cases showed IVC and HV involvement and 3 (7.5%) cases showed isolated IVC involvement. For location of IVC lesion, 21 (52.5%) cases showed diaphragm IVC involvement, and 19 (47.5%) cases showed retrohepatic IVC involvement. Thirty-eight (95.0%) patients had IVC thrombosis involvement. (Table 1). Twenty-three (57.5%) patients had previous medications for comorbidities, including gastrointestinal bleeding (n = 9), renal vein thrombosis (n = 4), lower limb venous thrombus (n = 5), hepatic encephalopathy (n = 3), hypertension (n = 1) and diabetes mellitus (n = 1). Gastrointestinal bleeding was the most common comorbidity, abdominal pain or distention was the most common symptom, and 22.5% of the patients had a history of trauma or surgery (Table 2).

**Table 1:** Patient characteristics of study population.

Parameters	Value
n	40
Sex, Male	32 (78.6%)
Median age, years (Range)	49.5 (28–76)
Median course of disease, months (IQR)	24 (1.2, 123)
Previous medications	23 (57.5%)
Lesion types	
IVC and HV involvement	37 (92.5%)
Isolated IVC involvement	3 (7.5%)
Location of IVC lesion	
Diaphragm IVC	21 (52.5%)
Retrohepatic IVC	19 (47.5%)
IVC thrombosis involvement	38 (95.0%)

IVC, Inferior vena cava; HV, Hepatic vein.

**Table 2:** Comorbidities and symptoms of patients with IVC thrombosis.

Comorbidities	No. (%)	Symptoms	No. (%)
Gastrointestinal bleeding	9 (22.5%)	Abdominal Pain/distention	25 (62.5%)
Ulcer of lower limb	4 (10.0%)	Varices in abdomin and legs	11 (27.5%)
History of trauma/surgery	9 (22.5%)	Lower limb edema/pain	18 (45.0%)
Renal vein thrombosis	4 (10.0%)	Lower-extremity pigmentation	10 (25.0%)
Lower limb venous thrombus	5 (12.5%)	Ulcer of lower limb	4 (10.0%)
Hepatic encephalopathy	3 (7.5%)	Haematemesis/hematochezia	9 (22.5%)
Hepatocarcinoma	1 (2.5%)	Edema of scrotum	2 (5.0%)
Hypertension	1 (2.5%)	Obnubilation/drowsiness	3 (7.5%)
Diabetes mellitus	1 (2.5%)	Lacking in strength	5 (12.5%)
Others (hepatic failure, etc)	5 (12.5%)	Loss of appetite	5 (12.5%)

### Procedural results and perioperative complications

Recoverable stents placement, balloon angioplasty and thrombolysis were technically successful in all patients. IVC was patent immediately after recoverable stents placement by IVC angiography, and all thrombus in IVC had completely resolved before recoverable stents removal. Only one recoverable stents was placed for each patient, and recoverable stents were retrieved 8 to 29 days after placement, with a median duration of 24.0 days (IQR 1.2, 135.0). There were no adverse events related to the balloon dilation during the procedure and thrombolysis. Only one death was procedure-related, a patient who died from acute pulmonary thromboembolism 6 hours after recoverable stents placement. The overall survival rate was 97.5% (39/40) perioperatively. Except for two patients who died after leaving the hospital, recoverable stents removals were technically successful in 36 patients. Only one recoverable stents fractured during a removal attempt, which was eventually extracted surgically, yielding a technical success rate of 92.3% (36/39). Recoverable stents migration occurred in one patient, and insufficient expansion of recoverable stents was found in two patients, who were successfully dilated by balloon angioplasty. One patient developed rigors just after intervention and symptom were relieved 20 minutes later after oxygen uptake and injection of dexamethasone. Dyspnea was found in one patient due to pleural effusion and this symptom disappeared after effective drainage. One patient developed acute cerebral infarction during hospitalization, but recovered and was discharged after treatment (Table 3).

**Table 3:** Interventional procedures and perioperative complications.

Procedures and complications	Value
Interventional procedures	
Balloon angioplasty of IVC	34 (85.0%)
Agitation thrombolysis of IVC	13 (32.5%)
Catheter-directed thrombolysis of IVC	11 (27.5%)
Balloon dilation of HV	6 (15.0%)
Stent placement of HV	1 (2.5%)
Gastric coronary vein embolization	8 (20.0%)
TIPSS	3 (7.5%)
Z-expandable metallic stent	1 (2.5%)
Retrival time of stents, days (range)	14.6 ± 4.9 (8–29)
Perioperative complications	
Acute pulmonary thromboembolism	1 (2.5%)
Failure retrival of stent filter	1 (2.5%)
Migration of stent filter	1 (2.5%)
Insufficient expansion of stent filter	2 (5.0%)
Dyspnea due to pleural effussion	1 (2.5%)
Shiver with cold or fear	1 (2.5%)
Acute brain faction	1 (2.5%)

IVC, Inferior vena cava; HV, Hepatic vein; TIPSS, Transjugular intrahepatic portosystemic stent shunt.

### Color Doppler ultrasound results

**Figure 5:**
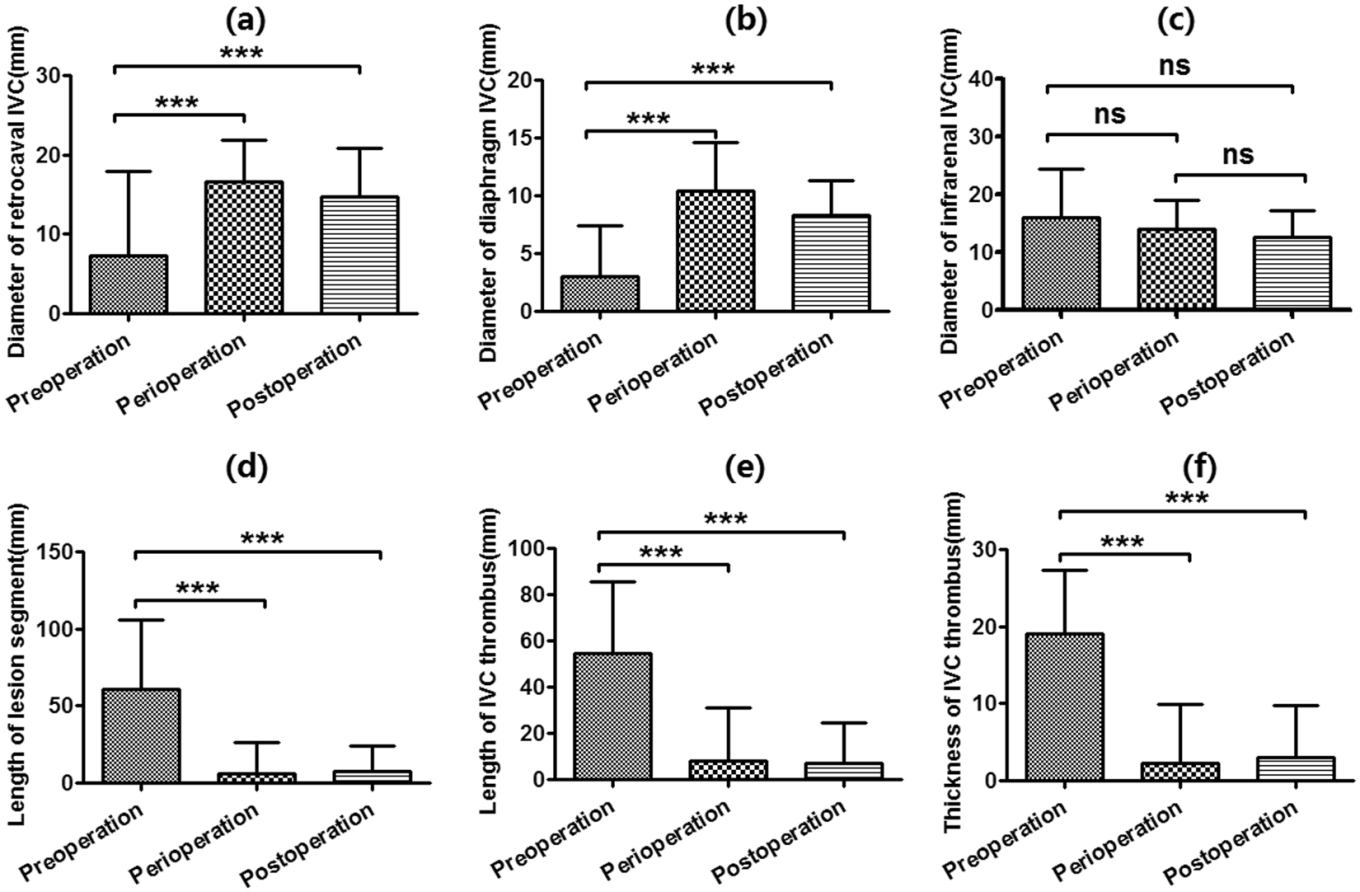
Results of color Doppler ultrasound test. The diameter of retrocaval IVC (**a**) and diaphragm IVC increased (**b**) compared to the diameter prior to intervention. The diameter of infrarenal IVC (**c**) did not changed significantly. The length of lesion segment (**d**), length (**e**) and thickness of IVC thrombus (**f**) decreased significantly. ***p < 0.0001, ns = not significant.

Compared to diameter prior to intervention, the mean diameter of retrocaval IVC increased from 8.0 ± 10.7 mm to 16.2 ± 5.3 mm before recoverable stents retrieval (*p* < 0.0001), and to 14.8 ± 5.8 mm after intervention (*p* < 0.0001). The mean diameter of diaphragm IVC increased from 3.2 ± 4.4 mm to 10.1 ± 4.0 mm before recoverable stents retrieval (*p* < 0.0001), and to 8.2 ± 2.9 mm after intervention (*p* < 0.0001). The mean diameter of infrarenal IVC did not change significantly (*p* > 0.05). The mean length of lesion segment decreased significantly both perioperatively and postoperatively (*p* < 0.0001). The mean length and thickness of IVC thrombus also decreased significantly perioperatively and postoperatively (*p* < 0.0001). The mean retrocaval IVC was almost occluded with a mean blood flow rate of 4.9 ± 11.9 cm/s before intervention. Blood flow restored patency and significantly increased to a mean of 78.0 ± 45.3 cm/s perioperatively, and to 90.6 ± 35.9 cm/s postoperatively (*p* < 0.0001, Fig. 5).

### Follow-Up

With the exception of one patient who died perioperatively, these remaining 39 patients were followed-up (median 43.7 months; IQR 3.9, 84.7 months; range, 0.1–139.6 months), and 5 patients (12.8%) were lost to follow-up. There were 3 deaths within one month, including the one patient who died 6 hours after procedure, for a hospital mortality of 7.5% (3/40). Two patients died from hepatic failure 0.8 and 5.4 months after procedure, one patient died from hepatocarcinoma after 101.8 months, and one patient died from pulmonary embolism after 0.4 month. The 5-year and 10-year survival rates were 89.5% and 67.1%. One patient was found to have developed reobstruction of the IVC without thrombosis 130.2 months after the procedures due to recoil at the angioplasty site, and subsequently underwent Z-expandable metallic stent placement. One patient showed restenosis after 10.6 months without any treatment. The primary and secondary patency rate at final follow-up visits was 94.3% (33/35) and 97.1% (34/35), respectively.

## Discussion

The primary BCS is relatively common in Oriental countries and South Africa, such as Japan, China, South Africa and India, which is well known as membranous obstruction of IVC^7,9,13,14^. The secondary BCS is most common in Western countries. Thrombus formation is a common complication of membranous obstruction of IVC, which occurring in 28.6% of patients^14^. Balloon angioplasty or stent placement has been considered successful treatment for IVC obstruction^4,7^. However, treatment of patients with BCS complicated by IVC thrombosis remains challenging because precluded balloon angioplasty or stent placement is precluded due to the risk of acute fatal pulmonary thromboembolism after mechanically reestablishing IVC patency. Since the first successful use of thrombolysis with streptokinase in acute BCS, different agents including urokinase and tissue plasminogen activator have been used to dissolve the thrombus^15^. Except for thrombolysis, other therapies were considered the treatments of choice. Ishiguchi *et al*.^10^ reported their treatment of BCS complicated by thrombosis with combinations of thrombolysis and stent placement. Although most patients can been successfully managed with local thrombolysis, angioplasty, and stents^10^ and angioplasty has been able to relieve symptoms in more than 70% of patients, stent placement carries the risk of restenosis or occlusion of hepatic vein or accessory hepatic veins or both. Approximately 13% of cases who underwent stent placement still developed restenosis^7^. Follow-up by Doppler ultrasonography was needed to determine the patency of IVC^7,11,12^.

Recoverable stents were used in this study for the treatment of patients with BCS complicated by IVC thrombosis to overcome the aforementioned problems. The Z-stents served to compress the thrombosis and expanded the obstructive IVC and maintains its patency. Its retrieval is beneficial to avoid long-term complications caused be permanent stent, including stent migration, occlusion of hepatic veins or accessory hepatic veins, restenosis or occlusion of IVC, and so forth. The results of the current study, including a 100% placement rate, complete resolution of thrombi, a 97.3% retrieval rate, and a 94.3% primary patency rate are favorable than any previously reported^7,11,12^. Significantly, the diameter of retrocaval IVC and diaphragm IVC increased and the length and thickness of IVC thrombus decreased after treatment. A few serious complications, including one acute pulmonary thromboembolism, one migration, and one failure retrieval of recoverable stents occurred. The long-term results were satisfactory in all but 2 patients who presented with a restenosis or reobstruction and underwent additional therapy. A retrieval hook lies at the apex of the cone and its attached legs were used for retrieval and prevention of pulmonary embolism. There were 5 deaths during the study period, and only one patient died owing to pulmonary embolism, and the remained 4 deaths were unrelated to thrombotic risks. Our data illustrate the safety and effectiveness of the recoverable stents for managing BCS patients complicated with IVC thrombosis.

However, this study has several limitations. This was a single-center study with no controls. This was only a retrospective case analysis with a relatively small sample size. In addition, although this was a long-term study, a lost to follow-up rate of 12.8% is relatively high. Besides, previous medical conditions including anticoagulant therapymight have impact on venous thrombosis. In conclusion, recoverable stents treatment is safe and effective for BCS complicated by IVC thrombosis, and is associated with a good long-term outcome.
